# Impaired Glymphatic Function in Acute Spontaneous Intracerebral Hemorrhage

**DOI:** 10.1111/cns.70252

**Published:** 2025-02-06

**Authors:** Jinsong Cai, Yecheng Dong, Mengmeng Fang, Kai Wei, Shenqiang Yan, Ying Zhou

**Affiliations:** ^1^ Department of Radiology The Second Affiliated Hospital of Zhejiang University, School of Medicine Hangzhou China; ^2^ Department of Neurology The Second Affiliated Hospital of Zhejiang University, School of Medicine Hangzhou China; ^3^ State Key Laboratory of Transvascular Implantation Devices The Second Affiliated Hospital of Zhejiang University, School of Medicine Hangzhou China

**Keywords:** diffusion tensor imaging, glymphatic system, intracerebral hemorrhage

## Abstract

**Background and Aims:**

Alterations in glymphatic function during the acute phase of acute spontaneous intracerebral hemorrhage (sICH) remain poorly understood. The aim of this study was to investigate whether, compared to healthy controls (HCs), the glymphatic system is impaired in patients with sICH, and to assess its association with hemorrhage and edema severity and outcome.

**Methods:**

Fifty‐five sICH patients (including 46 supratentorial sICH and 9 subtentorial sICH) and 97 age‐ and sex‐matched HCs underwent conventional MRI and diffusion tensor imaging. The diffusion along the perivascular space (DTI‐ALPS) index, serving as a marker for glymphatic function, was computed, with supratentorial cases being categorized into ipsilateral and contralateral ALPS. Volumes of hemorrhage and edema were evaluated using susceptibility‐weighted imaging (SWI) and T2‐weighted magnetic resonance images, and the relative edema ratio was calculated. Clinical outcomes were categorized as favorable or poor based on a modified Rankin scale score of ≤ 2 or > 2 at 90 days.

**Results:**

sICH patients showed significantly lower DTI‐ALPS values on the ipsilateral side compared to the average in the HC group (1.34 ± 0.24 vs. 1.46 ± 0.22, *p* = 0.003), whereas contralateral DTI‐ALPS values in sICH patients did not differ significantly from HCs (1.48 ± 0.21 vs. 1.46 ± 0.22, *p* = 0.524). The ipsilateral DTI‐ALPS was notably associated with both hemorrhage and relative edema volumes (both *p* < 0.05). A higher ipsilateral DTI‐ALPS was independently associated with a favorable outcome at 90 days (odds ratio = 1.686 per 0.1 increase, *p* = 0.038).

**Conclusions:**

The DTI‐ALPS index, which reflects glymphatic functionality, is notably diminished on the ipsilateral side in acute sICH, correlating significantly with increased volumes of hemorrhage and edema. This study suggests that glymphatic dysfunction may contribute to the severity of clinical outcomes, and highlights the potential role of the glymphatic system in the pathophysiology of sICH.

## Graphical abstract

This study demonstrates that the DTI‐ALPS index, which reflects glymphatic functionality, is significantly diminished on the ipsilateral side in patients with acute spontaneous intracerebral hemorrhage. Lower DTI‐ALPS values are associated with increased volumes of hemorrhage and edema as well as poor clinical outcomes at 90 days.

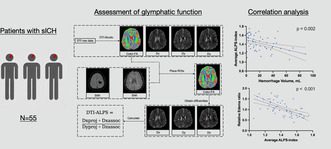

## 1 Introduction

Spontaneous intracerebral hemorrhage (sICH), the most common type of hemorrhagic stroke, accounts for approximately 15% of all stroke incidents and is associated with a high morbidity and mortality rate, nearing 40% [1, 2]. Despite advancements in the understanding of prognostic factors, risk elements, and the neural tissue's response to hematoma accumulation [3], outcomes for patients remain predominantly unfavorable. Presently, treatment modalities are mainly supportive, with surgical options reserved for cases presenting significant mass effect, herniation, or hydrocephalus [4]. Furthermore, survivors of sICH are at continued risk for delayed neurological injury, driven by inflammatory and cytotoxic responses to the hematoma and its degradation byproducts [5]. This recognition has propelled secondary brain injury to the forefront of therapeutic investigation within the intracerebral hemorrhage research community [6, 7]. Perihematomal edema, indicative of secondary damage, is particularly significant, reflecting the combined effects of thrombin build‐up, the influx of inflammatory mediators, and erythrocyte breakdown [8, 9]. Ongoing research is dedicated to deepening the understanding of intracerebral hemorrhage pathophysiology with the aim of pinpointing viable therapeutic targets.

The discovery of the glymphatic system by Iliff et al. marked a significant advancement in our comprehension of cerebral waste clearance mechanisms [10]. This system integrates arterial conduits, aquaporin‐4 (AQP4) water channels on astroglial endfeet, and the surrounding perivascular spaces, facilitating the flow of cerebrospinal fluid (CSF) and interstitial fluid, thereby enhancing the removal of metabolic wastes. The entry of CSF into the periarterial spaces enables an exchange with interstitial fluid, assisting in the clearance of amyloid‐beta, tau proteins, and other metabolites, as well as proinflammatory factors. Ultimately, these wastes are directed toward lymphatic drainage sites [11]. Notably, the dilatation of perivascular pathways, commonly observed in various pathologies, is critical to the efficacy of this clearance process.

In the realm of cerebrovascular disorders, dysfunction of the glymphatic system plays a critical role in a multitude of detrimental processes, including edema, disruption of the blood–brain barrier, and neuroinflammatory responses [12]. Notably, an observed expansion of perivascular spaces in cases of intracerebral hemorrhage correlates with impaired glymphatic waste clearance, a phenomenon similarly noted in conditions such as traumatic brain injury [13, 14]. Experimental data from rodent studies demonstrate that obstructing glymphatic drainage exacerbates brain edema, inflammation, and neuronal apoptosis, which contribute to neurological impairments in intracerebral hemorrhage. This is mediated by alterations in AQP‐4, tumor necrosis factor‐alpha (TNF‐α), and interleukin‐10 (IL‐10) levels, thereby highlighting the glymphatic system's protective role under physiological conditions [15].

A recent study employing noninvasive diffusion tensor imaging to assess the perivascular space (DTI‐ALPS) identified glymphatic dysfunction on the ipsilateral side of lesions in chronic phase sICH patients [16]. However, alterations in glymphatic function during the acute phase of sICH remain poorly understood. Therefore, the present study aims to evaluate glymphatic function utilizing DTI‐ALPS in patients with acute sICH, investigate the factors that influence this function, and explore its association with the severity of hemorrhage and edema, as well as with clinical outcomes.

## 2 Methods

### 2.1 Patients

The study was conducted in accordance with the Helsinki Declaration and received approval from the Ethics Committee of the Second Affiliated Hospital of Zhejiang University, School of Medicine (approval number: Yan‐20,240,539). Written informed consent was obtained from all subjects or their legal representatives prior to participation. We recruited a convenience sample of 55 hospitalized patients diagnosed with sICH from May 2023 to February 2024, all of whom received conservative management. The inclusion criteria included: (1) age ≥ 18 years, (2) verified diagnosis of intraparenchymal hemorrhage by CT or MRI scan, and (3) noncomatose status both 1 week prior to and following MRI administration. Exclusion criteria were established as follows: (1) accompanied by intraventricular hemorrhage or subarachnoid hemorrhage; (2) MRI detection of brain parenchymal lesions other than hemorrhage, (3) secondary causes of ICH such as hemorrhagic transformation of ischemic stroke, aneurysmal, cavernomas, arteriovenous malformations, central venous thrombosis, trauma‐related, or tumor, (4) surgical evacuation of hematoma, (5) excessively large hemorrhagic lesions that could interfere with the measurement of DTI metrics, or (6) history of neuropsychiatric disorders. A cohort of 97 healthy volunteers was selected to serve as healthy controls (HCs). Their inclusion criteria were: (1) age over 40; (2) no history of significant cerebrovascular events except lacunar infarction; (3) absence of infarct lesions with restricted diffusion on current diffusion‐weighted imaging; (4) absence of intracranial hemorrhage on current susceptibility‐weighted imaging; (5) no history of multiple sclerosis, Alzheimer's disease, Parkinson's disease, or head trauma; (6) absence of white matter lesions of nonvascular origin such as immunological‐demyelinating, metabolic, toxic, or infectious causes. All participants underwent multimodal MRI. Demographic information and vascular risk factors such as age, gender, and history of hypertension, diabetes, hyperlipidemia, and smoking were recorded. The modified Rankin Scale (mRS) score was recorded 90 days following the onset of hemorrhage. Clinical outcomes were defined as favorable or poor based on a mRS of ≤ 2 or > 2, respectively.

### 2.2 MRI Protocol

The image acquisition was performed with on a 3.0 T MRI using the system's 20‐channel head array coil. DTI images were obtained using a Single‐SE diffusion‐weighted echo‐planar imaging (EPI): TR/TE = 5000/77 ms, FOV = 220 × 220 mm, matri = 128 × 128, *b* = 1000 s/mm^2^, voxel size = 1.7 × 1.7 × 2 mm^3^, 30 directions, thickness = 2 mm, gap = 0.4 mm. Susceptibility weighted imaging (SWI) using 3D gradient recalled echo and minimum intensity projection (mIP) images: TR/TE = 28/20 ms, FOV = 220 × 200 mm, matrix = 320 × 307, voxel size = 0.7 × 0.7 × 2 mm^3^, thickness = 2 mm without slice gap. T2‐weighted TSE: TR/TE = 3800/99 ms, FOV = 230 × 200 mm, matrix = 384 × 320, voxel size = 0.6 × 0.6 × 6 mm^3^, thickness = 6 mm, gap = 1.2 mm.

### 2.3 Volume Assessments of Hemorrhage and Edema

SWI and T2‐weighted images were analyzed using MRIcron software (available at http://www.nitrc.org/projects/mricron). Hematomas were delineated on SWI images, while brain edema was identified on T2‐weighted images by subtracting the volume of hematomas from the total lesion volume. The imaging analysis involved a collaborative effort between an experienced radiologist (C.J.) and a seasoned neurologist (Y.S.), both of whom were blinded to clinical and additional imaging data. Manual corrections were performed jointly, and the final delineation of lesions was established through their consensus. Then the relative edema ratio was determined by calculating the quotient of the edema volume to the hemorrhage volume. The site of the hematoma was categorized as supratentorial (including lobar and deep) or subtentorial.

### 2.4 Calculation of DTI‐ALPS

**FIGURE 1:**
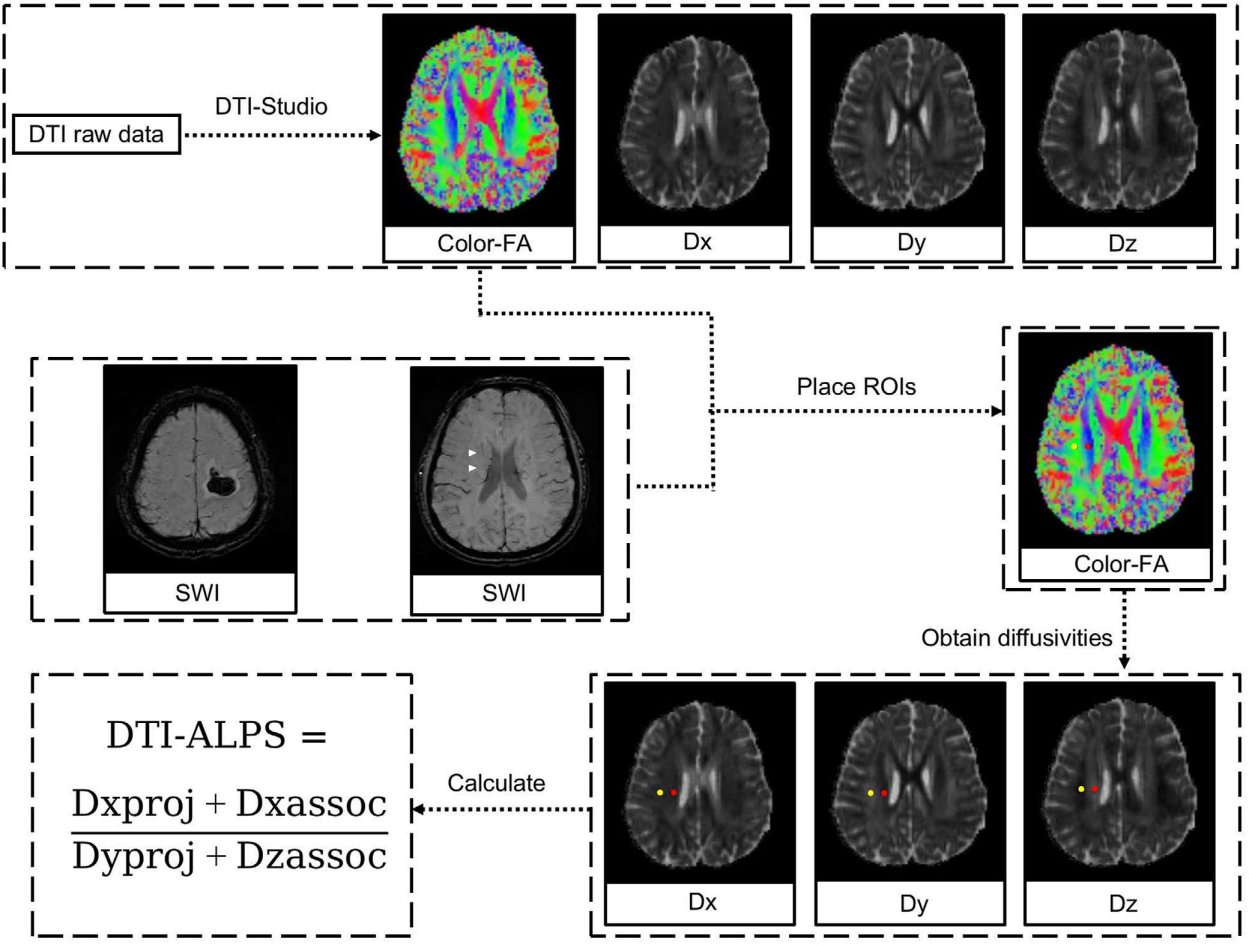
Workflow for Processing the Diffusion Tensor Imaging Along the Perivascular Space (DTI‐ALPS) Index. Color‐coded fractional anisotropy (FA) maps were generated from diffusion tensor imaging (DTI) raw data. At locations corresponding to deep medullary veins (DMVs), indicated by white triangular arrows on the susceptibility weighted imaging (SWI) and oriented perpendicular to the body of the ventricle, two regions of interest (ROIs) were established on the color‐FA map. These ROIs were positioned within the projection fibers, marked by a red circle, and the association fibers, denoted by a yellow circle, on the color‐FA image. The diffusivities of ROIs in the directions of the x‐axis (Dx), y‐axis (Dy) and z‐axis (Dz) were obtained accordingly. The DTI‐ALPS index was then calculated as [(Dxproj + Dxassoc)/(Dyproj + Dzassoc)].

Evaluation of the ALPS index was conducted in accordance with methodologies outlined in previous studies [17, 18, 19]. Diffusivity maps along the x‐axis (Dx), y‐axis (Dy), z‐axis (Dz), and color‐coded fractional anisotropy (FA) maps were processed using DTI Studio (available at https://www.mristudio.org). Two regions of interest (ROIs), each 5 mm in diameter, were positioned on the color‐coded FA map specifically within the projection fibers and the association fibers, located where the direction of the deep medullary veins (DMVs) was perpendicular to the body of the ventricle. The diffusivities in the Dx, Dy, and Dz directions were recorded for each ROI within the projection fibers (Dx_proj, Dy_proj, Dz_proj) and the association fibers (Dx_assoc, Dy_assoc, Dz_assoc), respectively. The ALPS index was then calculated using the formula [(Dx_proj + Dx_assoc) / (Dy_proj + Dz_assoc)]. For patients with supratentorial ICH, cases were categorized into ipsilateral and contralateral ALPS. Figure 1 provides a schematic illustration of the method used for measuring diffusivity with the ALPS index.

### 2.5 Statistical Analysis

Statistical analyses were performed using IBM SPSS 19.0. The Kolmogorov–Smirnov test was used to assess the distribution patterns of the data. Continuous variables with normal distributions are presented as means ± standard deviations (SD), while other variables are reported as medians with interquartile ranges (IQR). Categorical data are expressed as frequencies (proportions). Group comparisons were conducted using the Student's *t*‐test after confirmation of data normality. Correlations between groups were assessed using either Pearson's or Spearman's correlation tests, depending on the data distribution. Multivariate analyses, including linear and logistic regression, were performed by incorporating factors with a *p*‐value < 0.1 and significant covariates from previously published studies. A *p* value of < 0.05 was considered statistically significant.

## 3 Results

### 3.1 Demographic Characteristics of sICH and HC Participants

In this study, we included 55 sICH patients (25.5% females; mean age = 58 ± 15 years) and 97 age‐ and sex‐matched healthy controls (HCs) (25.8% females; median age = 58 ± 7 years). The average duration of the disease among sICH patients was 5.3 ± 3.1 days. Hemorrhage volumes measured an average of 22.1 ± 20.1 mL, and edema volumes averaged 24.2 ± 26.2 mL. Among them, 46 patients had supratentorial intracerebral hemorrhage, including 16 cases of lobar hemorrhage and 30 cases of deep hemorrhage, while 9 patients had subtentorial intracerebral hemorrhage. Additionally, none of the supratentorial patients had bilateral hemorrhage. The DTI‐ALPS on the ipsilateral side was significantly lower in the sICH group compared to the HC group (1.34 ± 0.24 vs. 1.46 ± 0.22, *p* = 0.003). However, there was no significant difference in DTI‐ALPS between the contralateral sides of the sICH and HC groups (1.48 ± 0.21 vs. 1.46 ± 0.22, *p* = 0.524). Table 1 presents a comparison of demographic and clinical data between the sICH and HC groups.

**TABLE 1:** Demographic and Clinical Profiles of the spontaneous intracerebral hemorrhage (sICH) and healthy control (HC) Groups.

	HC (*n* = 97)	sICH (*n* = 55)	*p*
Age	58 ± 7	58 ± 15	0.916
Female	25 (25.8%)	14 (25.5%)	1.000
Hypertension	64 (66.0%)	40 (72.7%)	0.469
Diabetes	17 (17.5%)	7 (12.7%)	0.495
Hyperlipemia	22 (22.7%)	5 (9.1%)	0.046
Smoking	25 (25.8%)	14 (25.5%)	1.000
Ipsilateral DTI‐ALPS (*n* = 46)	1.45 ± 0.22	1.34 ± 0.24	0.003
Contralateral DTI‐ALPS (*n* = 46)	1.45 ± 0.22	1.48 ± 0.21	0.524
Average DTI‐ALPS	1.45 ± 0.22	1.42 ± 0.17	0.336

Abbreviation: DTI‐ALPS, diffusion along the perivascular space.

### 3.2 Determinants of Ipsilateral and Contralateral DTI‐ALPS

**FIGURE 2:**
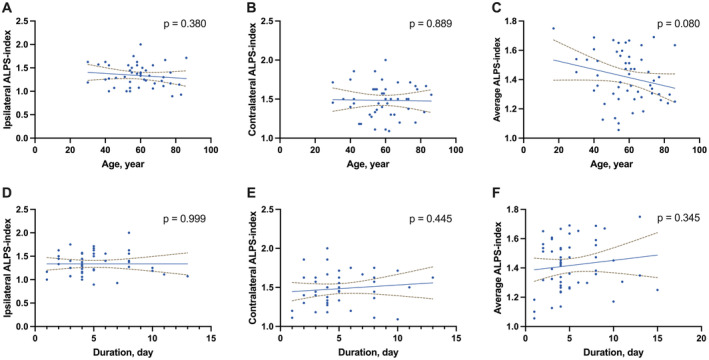
Scatter Plots of the Diffusion Along the Perivascular Space (DTI‐ALPS) Against Age and Disease Duration Across Conditions. (A–C) DTI‐ALPS plotted against age (years) for ipsilateral, contralateral, and average measurements, with *p*‐values of 0.380, 0.889, and 0.080, respectively. (D–F) DTI‐ALPS plotted against the duration of the condition (days) for ipsilateral, contralateral, and average measurements, with *p*‐values of 0.999, 0.445, and 0.345, respectively. Each plot represents individual observations as blue dots. The solid line in each graph denotes the linear regression line, while the dashed lines indicate the 95% confidence intervals. The *p*‐values above each plot assess the null hypothesis that there is no correlation between the variables; the higher values suggest a lack of statistically significant correlation in this sample.

The DTI‐ALPS value on the ipsilateral side was significantly lower compared to the contralateral side within the sICH group (1.34 ± 0.24 vs. 1.48 ± 0.21, *p* = 0.003). No significant differences were observed in DTI‐ALPS values, both ipsilateral and contralateral, between genders or among patients with or without hypertension, diabetes, hyperlipidemia, or smoking history (all *p* > 0.05, Table 2). Additionally, there was no significant correlation between age or disease onset duration and DTI‐ALPS on either side (all *p* > 0.05, Figure 2).

**TABLE 2:** Determinants of the Ipsilateral and Contralateral Diffusion Along the Perivascular Space (DTI‐ALPS).

	Yes	No	*p*
Ipsilateral DTI‐ALPS			
Female	1.39 ± 0.22	1.32 ± 0.25	0.463
Hypertension	1.31 ± 0.22	1.41 ± 0.32	0.256
Diabetes	1.25 ± 0.22	1.35 ± 0.25	0.378
Hyperlipemia	1.29 ± 0.19	1.34 ± 0.25	0.714
Smoking	1.32 ± 0.19	1.34 ± 0.26	0.532
Contralateral DTI‐ALPS			
Female	1.51 ± 0.12	1.48 ± 0.23	0.717
Hypertension	1.50 ± 0.22	1.44 ± 0.21	0.405
Diabetes	1.49 ± 0.24	1.48 ± 0.21	0.967
Hyperlipemia	1.47 ± 0.22	1.48 ± 0.22	0.925
Smoking	1.42 ± 0.16	1.51 ± 0.23	0.325

### 3.3 Impact of Ipsilateral and Contralateral DTI‐ALPS on Severity of Hemorrhage and Edema

**FIGURE 3:**
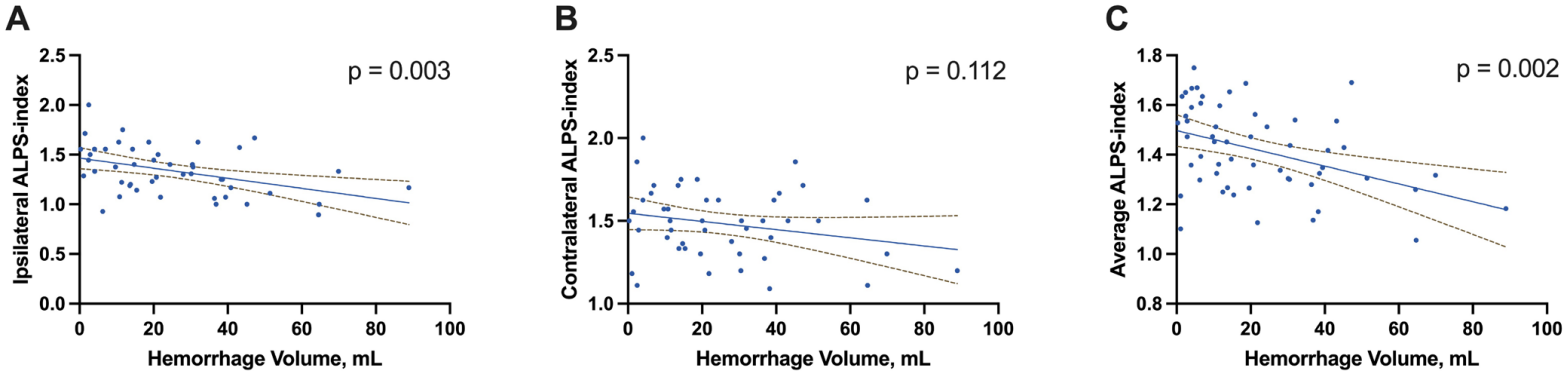
Association between the Diffusion Along the Perivascular Space (DTI‐ALPS) and Hemorrhage. The relationship between DTI‐ALPS measured ipsilaterally (A), contralaterally (B), and as an average (C) with the volume of hemorrhage in milliliters (mL). Blue dots represent individual patient data. Solid lines indicate the linear regression fit to the data, with dashed lines depicting the 95% confidence intervals for the fit. *p*‐values are provided for each correlation, with none of the panels showing a statistically significant correlation (*p* < 0.05).

**FIGURE 4:**
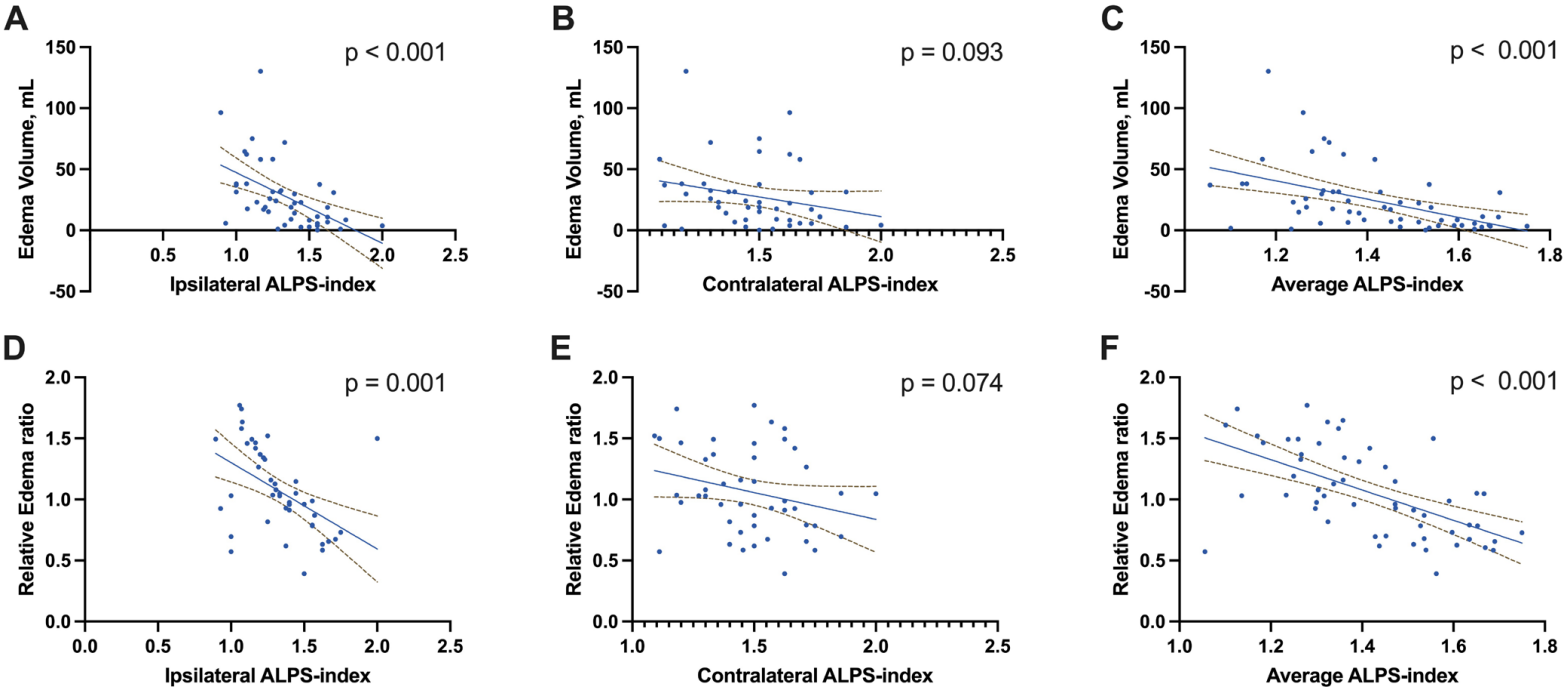
Association between the Diffusion Along the Perivascular Space (DTI‐ALPS) and Edema. The relationship between DTI‐ALPS measured ipsilaterally (A), contralaterally (B), and as an average (C) with the volume of edema in milliliters (mL). The relationship between DTI‐ALPS measured ipsilaterally (D), contralaterally (E), and as an average (F) with the relative edema ratio. Blue dots represent individual patient data. Solid lines indicate the linear regression fit to the data, with dashed lines depicting the 95% confidence intervals for the fit. *p*‐values are provided for each correlation, with none of the panels showing a statistically significant correlation (*p* < 0.05).

Figure 3 demonstrates that the ipsilateral DTI‐ALPS was significantly associated with hemorrhage volumes (*r* = −0.426, *p* = 0.003). Figure 4 shows that both the edema volume and the relative edema ratio were significantly correlated with the ipsilateral DTI‐ALPS (*r* = −0.592, *p* < 0.001; *r* = −0.489, *p* = 0.001). No other factors showed significant correlations with either hemorrhage or edema volumes (all *p* > 0.05, Table 3).

**TABLE 3:** Univariate analysis for Hemorrhage and Edema Volumes.

	Yes	No	*p*
Hemorrhage and volume			
Female	17.3 ± 16.1	23.7 ± 21.2	0.308
Hypertension	21.9 ± 17.0	22.5 ± 27.4	0.921
Diabetes	25.0 ± 21.9	21.7 ± 20.0	0.684
Hyperlipemia	14.9 ± 20.7	22.8 ± 20.1	0.410
Smoking	19.7 ± 18.5	22.9 ± 20.8	0.613
Edema volume			
Female	21.0 ± 22.8	25.3 ± 27.5	0.601
Hypertension	23.6 ± 20.5	25.8 ± 38.5	0.785
Diabetes	32.4 ± 33.6	23.0 ± 25.2	0.382
Hyperlipemia	20.7 ± 30.8	24.6 ± 26.1	0.755
Smoking	18.7 ± 19.7	26.1 ± 28.1	0.366

	Pearson r	*p*
Hemorrhage and volume		
Age, year	0.100	0.468
Duration, day	−0.154	0.260
Edema volume		
Age, year	0.212	0.120
Duration, day	−0.041	0.767

### 3.4 Influence of Ipsilateral DTI‐ALPS on Clinical Outcomes in sICH Patients

A total of 39 patients (70.9%) with sICH exhibited favorable clinical outcomes at 90 days postonset. In comparison to those with poor outcomes, patients with favorable outcomes were younger, with smaller volumes of hemorrhage and edema, lower baseline National Institutes of Health Stroke Scale (NIHSS) scores, and lower DTI‐ALPS on the ipsilateral side in 46 patients with supratentorial ICH (all *p* < 0.05, Table 4). After adjusting for age, sex, and baseline NIHSS scores, a higher ipsilateral DTI‐ALPS was found to be independently predictive of a favorable outcome at 90 days (odds ratio = 1.686 per 0.1 increase, *p* = 0.038, Table 5). For the 9 patients with subtentorial ICH, no significant difference in the average DTI‐ALPS was observed between those with a favorable outcome at 90 days and those without (*p* = 0.242).

**TABLE 4:** Clinical and Imaging Determinants of Clinical Outcomes.

	mRS 0–2 (*n* = 39)	mRS 3–6 (*n* = 16)	*p*
Age	54 ± 14	68 ± 13	0.001
Female	8 (20.5)	6 (37.5)	0.306
Hypertension	26 (66.7)	14 (87.5)	0.184
Diabetes	3 (7.7)	4 (25.0)	0.175
Hyperlipemia	4 (10.3)	1 (6.3)	1.000
Smoking	11 (28.2)	3 (18.8)	0.734
Basline NIHSS	3.6 ± 4.7	10.6 ± 3.8	< 0.001
Hemorrhage location			
Deep	20 (51.3)	10 (62.5)	0.500
Lobar	12 (30.8)	4 (25.0)	
Subtentorial	7 (17.9)	2 (12.5)	
Hemorrhage volume, mL	16.8 ± 15.4	34.9 ± 24.6	0.002
Edema volume, mL	15.6 ± 14.8	45.3 ± 35.4	< 0.001
Relative edema ratio	0.97 ± 0.36	1.26 ± 0.27	0.005
Ipsilateral DTI‐ALPS	1.41 ± 0.24	1.16 ± 0.16	0.001
Contralateral DTI‐ALPS	1.51 ± 0.21	1.43 ± 0.22	0.290
Average DTI‐ALPS	1.47 ± 0.17	1.30 ± 0.08	0.001

Abbreviations: DTI‐ALPS, diffusion along the perivascular space; mRS, modified Rankin scale score; NIHSS, National Institutes of Health Stroke Scale.

**TABLE 5:** Binary Logistic Regression Analysis for Predicting Clinical Outcome (mRS 0–2).

	Odds ratio	95% confidence interval	*p*
Age	0.934	0.861, 1.014	0.101
Sex	0.251	0.026, 2.391	0.229
Baseline NIHSS score	0.833	0.679, 1.022	0.080
Hemorrhage volume, mL	0.975	0.923, 1.030	0.372
Ipsilateral DTI‐ALPS, per 0.1	1.686	1.030, 2.761	0.038

Abbreviations: DTI‐ALPS, diffusion along the perivascular space; mRS, modified Rankin scale score; NIHSS, National Institutes of Health Stroke Scale.

## 4 Discussion

The present study advances our understanding of glymphatic dysfunction in acute sICH, utilizing the DTI‐ALPS index as a novel biomarker. Our findings reveal a significant reduction in the DTI‐ALPS index on the ipsilateral side in sICH patients compared to healthy controls, highlighting disrupted glymphatic function that correlates with hemorrhage severity and edema progression. Notably, the impaired glymphatic clearance identified in our study suggests a potential mechanism linking hemorrhagic brain injury to subsequent edema and poorer clinical outcomes.

Glymphatic dysfunction in cerebrovascular diseases has been implicated in exacerbating secondary brain injury through mechanisms such as neuroinflammation and cytotoxic edema [12]. However, identifying glymphatic system biomarkers is challenging. The DTI‐ALPS provides a noninvasive method to assess glymphatic function, showing promise in neurological diseases like Alzheimer's disease and Parkinson's disease, neuroinflammatory diseases like multiple sclerosis and neuromyelitis optica spectrum disorder, and cerebral small vessel disease [19, 20, 21, 22]. In our study, the use of DTI‐ALPS in acute sICH revealed a significant decrease in glymphatic function on the affected side, highlighting its potential role in hemorrhage pathology. These findings are consistent with previous research on sICH patients with onset times exceeding 2 weeks [16]. Furthermore, the observed correlation between altered DTI‐ALPS indices on the lesion side, increased hemorrhage and edema volumes, and poorer clinical outcomes suggests that impaired glymphatic function may contribute to the severity of the condition. While this association does not establish causality, it supports the hypothesis that glymphatic dysfunction could be an important factor influencing the progression and recovery of brain injury following sICH. The glymphatic system, essential for the clearance of metabolic waste and neurotoxic substances, when impaired, may exacerbate the accumulation of hemorrhagic and edematous byproducts, potentially aggravating brain injury and influencing recovery trajectories.

The association between lower ipsilateral DTI‐ALPS values and unfavorable clinical outcomes at 90 days further positions this biomarker as a crucial prognostic tool. The extent of glymphatic dysfunction could serve as a biomarker for the severity of acute brain injury and potentially predict long‐term outcomes in sICH patients. Notably, our analysis detected no significant differences in DTI‐ALPS values across variables such as age, gender, or comorbidities, suggesting that post‐sICH glymphatic impairment is primarily driven by the hemorrhagic event rather than by individual predispositions. This insight underscores the utility of DTI‐ALPS as a diagnostic and prognostic tool that transcends demographic and clinical factors.

Moreover, the link between glymphatic dysfunction and clinical outcomes underscores the urgency for therapeutic strategies that enhance glymphatic function to mitigate secondary injury and improve recovery. This study found no correlation between the DTI‐ALPS index and disease duration, indicating a rapid decline in glymphatic functionality that does not promptly recover. Previous research shows that the DTI‐ALPS index increases over time in sICH patients whose symptoms began more than 2 weeks prior, suggesting a delayed recovery of glymphatic system function [16]. These findings imply that the glymphatic system may not effectively recover with conventional clinical treatments in the acute phase, potentially contributing to the challenges of effective intervention. Given the increasing research focus on enhancing glymphatic drainage [23, 24, 25, 26], improving glymphatic function could be a promising strategy to treat sICH, particularly in managing secondary edema posthemorrhage. This warrants further exploration into therapeutic interventions that can enhance glymphatic clearance and mitigate the impacts of cerebral hemorrhage.

Several limitations of this study need to be addressed. First, the sample size in our study is modest, particularly for subtentorial patients. The findings should be interpreted with caution and requires confirmation in a larger cohort. Second, while the DTI‐ALPS index, derived from perivascular space diffusion, serves as a proxy for glymphatic function, it may not fully correspond to the glymphatic activity confirmed through glymphatic MRI, despite its broad application in various diseases. Third, since glymphatic activity predominantly occurs during sleep, conducting scans during the daytime might not accurately capture complete glymphatic function. Fourth, this initial imaging study did not incorporate pathological or additional biomarkers, highlighting the necessity for more comprehensive future research to validate these findings. Future studies are needed to validate these findings across larger cohorts and to explore potential interventions that could modulate glymphatic function as part of the treatment regimen for sICH.

In conclusion, our study suggests that glymphatic dysfunction may play a significant role in the acute phase of sICH and is associated with clinical severity and outcomes. The DTI‐ALPS index presents a promising approach to improving our understanding of sICH pathophysiology and may help identify potential targets for therapies aimed at mitigating secondary brain injury. Further research is needed to establish a clearer cause‐effect relationship.

## Conflicts of Interest

The authors declare no conflicts of interest.

## Data Availability

The data that support the findings of this study are available from the corresponding author upon reasonable request.

## References

[1] V. L. Feigin , B. Norrving , and G. A. Mensah , “Global Burden of Stroke,” Circulation Research 120, no. 3 (2017): 439–448, 10.1161/CIRCRESAHA.116.308413. 28154096

[2] R. V. Krishnamurthi , A. E. Moran , M. H. Forouzanfar , et al., “The Global Burden of Hemorrhagic Stroke: A Summary of Findings From the GBD 2010 Study,” Global Heart 9, no. 1 (2014): 101–106, 10.1016/j.gheart.2014.01.003. 25432119

[3] C. D. Nouh , B. Ray , C. Xu , et al., “Quantitative Analysis of Stress‐Induced Hyperglycemia and Intracranial Blood Volumes for Predicting Mortality After Intracerebral Hemorrhage,” Translational Stroke Research 13, no. 4 (2022): 595–603, 10.1007/s12975-022-00985-x. 35040036

[4] S. M. Greenberg , W. C. Ziai , C. Cordonnier , et al., “2022 Guideline for the Management of Patients With Spontaneous Intracerebral Hemorrhage: A Guideline From the American Heart Association/American Stroke Association,” Stroke 53, no. 7 (2022): e282–e361, 10.1161/STR.0000000000000407. 35579034

[5] R. F. Keep , Y. Hua , and G. Xi , “Intracerebral Haemorrhage: Mechanisms of Injury and Therapeutic Targets,” Lancet Neurology 11, no. 8 (2012): 720–731, 10.1016/S1474-4422(12)70104-7. 22698888 PMC3884550

[6] M. Selim , L. D. Foster , C. S. Moy , et al., “Deferoxamine Mesylate in Patients With Intracerebral Haemorrhage (i‐DEF): A Multicentre, Randomised, Placebo‐Controlled, Double‐Blind Phase 2 Trial,” Lancet Neurology 18, no. 5 (2019): 428–438, 10.1016/S1474-4422(19)30069-9. 30898550 PMC6494117

[7] S. Urday , W. T. Kimberly , L. A. Beslow , et al., “Targeting Secondary Injury in Intracerebral Haemorrhage—Perihaematomal Oedema,” Nature Reviews Neurology 11, no. 2 (2015): 111–122, 10.1038/nrneurol.2014.264. 25623787

[8] A. F. Ducruet , B. E. Zacharia , Z. L. Hickman , et al., “The Complement Cascade as a Therapeutic Target in Intracerebral Hemorrhage,” Experimental Neurology 219, no. 2 (2009): 398–403, 10.1016/j.expneurol.2009.07.018. 19632224 PMC3731062

[9] W. C. Ziai , “Hematology and Inflammatory Signaling of Intracerebral Hemorrhage,” Stroke 44, no. 6 Suppl 1 (2013): S74–S78, 10.1161/STROKEAHA.111.000662. 23709738 PMC12054399

[10] J. J. Iliff , H. Lee , M. Yu , et al., “Brain‐Wide Pathway for Waste Clearance Captured by Contrast‐Enhanced MRI,” Journal of Clinical Investigation 123, no. 3 (2013): 1299–1309, 10.1172/JCI67677. 23434588 PMC3582150

[11] M. K. Rasmussen , H. Mestre , and M. Nedergaard , “The Glymphatic Pathway in Neurological Disorders,” Lancet Neurology 17, no. 11 (2018): 1016–1024, 10.1016/S1474-4422(18)30318-1. 30353860 PMC6261373

[12] T. Lv , B. Zhao , Q. Hu , and X. Zhang , “The Glymphatic System: A Novel Therapeutic Target for Stroke Treatment,” Frontiers in Aging Neuroscience 13 (2021): 689098, 10.3389/fnagi.2021.689098. 34305569 PMC8297504

[13] N. Bu , M. S. Khlif , R. Lemmens , et al., “Imaging Markers of Brain Frailty and Outcome in Patients With Acute Ischemic Stroke,” Stroke 52, no. 3 (2021): 1004–1011, 10.1161/STROKEAHA.120.029841. 33504185

[14] R. A. Opel , A. Christy , E. L. Boespflug , et al., “Effects of Traumatic Brain Injury on Sleep and Enlarged Perivascular Spaces,” Journal of Cerebral Blood Flow and Metabolism 39, no. 11 (2019): 2258–2267, 10.1177/0271678X18791632. 30092696 PMC6827121

[15] X. Liu , G. Wu , N. Tang , et al., “Glymphatic Drainage Blocking Aggravates Brain Edema, Neuroinflammation via Modulating TNF‐α, IL‐10, and AQP4 After Intracerebral Hemorrhage in Rats,” Frontiers in Cellular Neuroscience 15 (2021): 784154, 10.3389/fncel.2021.784154. 34975411 PMC8718698

[16] C. Zhang , J. Sha , L. Cai , et al., “Evaluation of the Glymphatic System Using the DTI‐ALPS Index in Patients With Spontaneous Intracerebral Haemorrhage,” Oxidative Medicine and Cellular Longevity 2022 (2022): 2694316, 10.1155/2022/2694316. 35847591 PMC9277160

[17] T. Taoka , Y. Masutani , H. Kawai , et al., “Evaluation of Glymphatic System Activity With the Diffusion MR Technique: Diffusion Tensor Image Analysis Along the Perivascular Space (DTI‐ALPS) in Alzheimer's Disease Cases,” Japanese Journal of Radiology 35, no. 4 (2017): 172–178, 10.1007/s11604-017-0617-z. 28197821

[18] H. Yokota , A. Vijayasarathi , M. Cekic , et al., “Diagnostic Performance of Glymphatic System Evaluation Using Diffusion Tensor Imaging in Idiopathic Normal Pressure Hydrocephalus and Mimickers,” Current Gerontology and Geriatrics Research 2019 (2019): 5675014, 10.1155/2019/5675014. 31320896 PMC6609364

[19] W. Zhang , Y. Zhou , J. Wang , et al., “Glymphatic Clearance Function in Patients With Cerebral Small Vessel Disease,” NeuroImage 238 (2021): 118257, 10.1016/j.neuroimage.2021.118257. 34118396

[20] H. Okazawa , M. Nogami , S. Ishida , et al., “PET/MRI Multimodality Imaging to Evaluate Changes in Glymphatic System Function and Biomarkers of Alzheimer's Disease,” Scientific Reports 14, no. 1 (2024): 12310, 10.1038/s41598-024-62806-5. 38811627 PMC11137097

[21] H. Pang , J. Wang , Z. Yu , et al., “Glymphatic Function From Diffusion‐Tensor MRI to Predict Conversion From Mild Cognitive Impairment to Dementia in Parkinson's Disease,” Journal of Neurology 271 (2024): 5598–5609, 10.1007/s00415-024-12525-8. 38913186 PMC11319419

[22] M. Kim , I. Hwang , J. H. Park , et al., “Comparative Analysis of Glymphatic System Alterations in Multiple Sclerosis and Neuromyelitis Optica Spectrum Disorder Using MRI Indices From Diffusion Tensor Imaging,” Human Brain Mapping 45, no. 5 (2024): e26680, 10.1002/hbm.26680. 38590180 PMC11002338

[23] S. Da Mesquita , A. Louveau , A. Vaccari , et al., “Functional Aspects of Meningeal Lymphatics in Ageing and Alzheimer's Disease,” Nature 560, no. 7717 (2018): 185–191, 10.1038/s41586-018-0368-8. 30046111 PMC6085146

[24] S. J. Hsu , C. Zhang , J. Jeong , et al., “Enhanced Meningeal Lymphatic Drainage Ameliorates Neuroinflammation and Hepatic Encephalopathy in Cirrhotic Rats,” Gastroenterology 160, no. 4 (2021): 1315–1329.e13, 10.1053/j.gastro.2020.11.036. 33227282 PMC7956141

[25] N. L. Hauglund , P. Kusk , B. R. Kornum , and M. Nedergaard , “Meningeal Lymphangiogenesis and Enhanced Glymphatic Activity in Mice With Chronically Implanted EEG Electrodes,” Journal of Neuroscience 40, no. 11 (2020): 2371–2380, 10.1523/JNEUROSCI.2223-19.2020. 32047056 PMC7083292

[26] S. Da Mesquita , Z. Papadopoulos , T. Dykstra , et al., “Meningeal Lymphatics Affect Microglia Responses and Anti‐Aβ Immunotherapy,” Nature 593, no. 7858 (2021): 255–260, 10.1038/s41586-021-03489-0. 33911285 PMC8817786

